# The Role of Virtual Clinics in Postoperative Total Knee Replacement Surgery Follow-Up during COVID-19 Pandemic

**DOI:** 10.1155/2022/9558511

**Published:** 2022-06-16

**Authors:** Endrotomo Sumargono, Maria Anastasia, Ifran Saleh, Erica Kholinne

**Affiliations:** ^1^St. Carolus Bone & Joint Center, St. Carolus Hospital, Jakarta, Indonesia; ^2^Faculty of Medicine, Universitas Katolik Indonesia Atma Jaya, Jakarta, Indonesia; ^3^Department of Orthopaedic and Traumatology, Faculty of Medicine, Universitas Indonesia, Jakarta, Indonesia; ^4^Faculty of Medicine, Universitas Trisakti, Jakarta, Indonesia

## Abstract

**Background:**

The purpose of this study was to evaluate the feasibility of the virtual clinic for outpatient follow-up care after TKR surgery.

**Methods:**

A total of 546 TKR surgeries were performed from January 2017 to December 2019. 30 patients were not able to go the hospital for routine follow-up. The data collections were taken for age, gender, year of surgery, functional score (Oxford Knee Score (OKS)), and active range of motion (ROM). The virtual clinic was conducted with the physician assistant and the operating surgeon via WhatsApp video call (WhatsApp Inc., Mountain View, California, USA) or Zoom (Zoom Video Communications, Inc., San Jose, California, USA).

**Result:**

The average follow-up period was 39.1 months. The earliest follow-up was 20 months, while the longest follow-up was 97 months. The average OKS score was 45. The average consultation time for the virtual clinic is 9 minutes 21 seconds. Most of the patients were satisfied with the online consultation, with only two patients having a satisfaction score below 80%.

**Conclusion:**

The virtual clinic for TKR surgery showed a high satisfaction rate during the COVID-19 pandemic, which has the potential to extend to the postpandemic era.

## 1. Introduction

The COVID-19 pandemic has led to the unusual practice of delivering healthcare services. During the COVID-19 pandemic, patients are more reluctant to come to the hospital. Furthermore, any elective outpatient clinic has been limited to free medical personnel and resources for patients with COVID-19 and to restrain COVID-19 transmission. Such a change has made many hospitals try any innovative solution, such as virtual clinics as a form of telemedicine [1]. Previously, in orthopedic outpatient clinics, telemedicine was underestimated. While a virtual orthopedic clinic lacks physical examination, the pandemic situation necessitates the utilization of any available resources [2]. A study from the United Kingdom has described that fracture virtual clinics can function as a triage center while providing follow-up care in 223 patients. Despite the limitation of the face-to-face appointment, the virtual clinic was reported to be well tolerated by patients and improved patient's safety and treatment [3, 4].

In our institution, joint arthroplasty is the most frequent orthopedic procedure, especially the total knee replacement (TKR). The challenge of running a joint replacement service becomes more evident since most of the patients are geriatric, which puts them at greater risk during the COVID-19 pandemic [5]. Due to the increasing number of COVID-19 cases in Indonesia, patients who underwent TKR surgery were concerned to visit the outpatient orthopedic clinics, while routine follow-up is important for the successful outcome of TKR surgery [6]. For this reason, we would like to surrogate the virtual fracture clinic to manage the TKR surgery outpatient orthopedic clinic. The purpose of this study was to assess the feasibility of the virtual clinic for outpatient follow-up care after TKR surgery.

## 2. Materials and Methods

**Figure 1:**
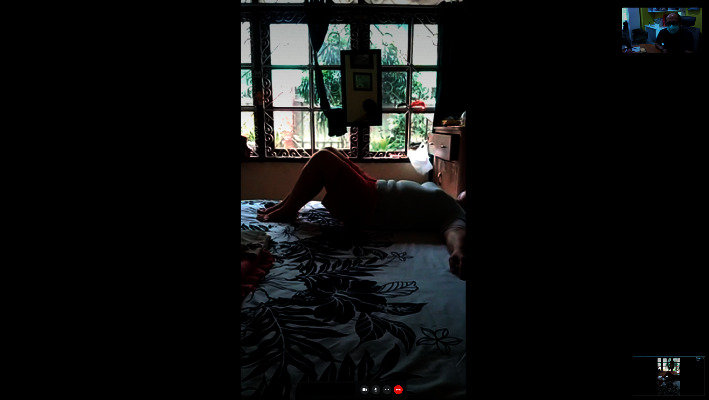
Patient's active ROM examined during WhatsApp video call and recorded as a screenshot.

The IRB was exempted from this study. A total of 546 TKR surgeries were performed from January 2017 to December 2019. The TKR implants used in all patients were from DePuy Synthes (Johnson and Johnson, New Brunswick, New Jersey, USA). The tracing of medical records found that 19 patients were deceased from unrelated causes. 497 patients were able to visit the clinic to attend the routine postoperative follow-up. However, 30 patients, who reside outside the city were not able to visit the hospital due to the concern from the pandemic and travel restriction from the Indonesian Government. These patients were contacted by the physician assistant and were offered a virtual follow-up clinic session with the operating surgeon via WhatsApp video call (WhatsApp Inc., Mountain View, California, USA) or Zoom (Zoom Video Communications, Inc., San Jose, California, USA) in May 2021 (Figure 1). The satisfaction rate towards TKR surgery was asked of the patient. Patients were informed that the video call would be recorded and clinical pictures would be taken. Verbal consent was obtained from each patient before the virtual clinic.

The data collection was taken for patient ID, age, gender, year of surgery, functional score, and active range of motion (ROM). Patients were given 12 questions from the Oxford Knee Score (OKS) questionnaire to evaluate their functional score [7]. Following the questionnaire, patients' ROM was observed by instructing them to perform knee extension to flexion in a supine position. The patient was given a video demonstration prior to it. The maximum flexion and extension of the knee joint were recorded. The patient's active ROM will be divided into two groups: <120 degrees and ≥120 degrees. Rowe et al. [8] reported that a minimum active ROM of 120° is a sufficient and suitable goal for the rehabilitation of knee motion. Descriptive statistics were utilized to report the patient's characteristics in the virtual follow-up clinic.

## 3. Results and Discussion

### 3.1. Results

**Figure 2:**
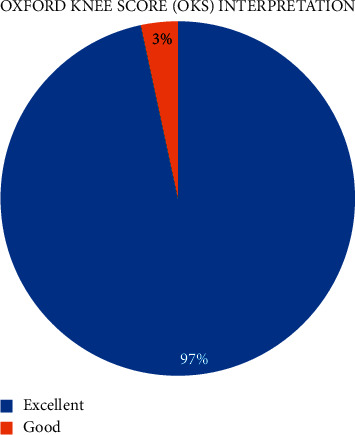
OKS interpretation.

**Figure 3:**
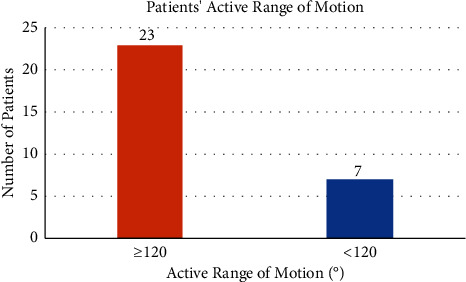
Range of motions among virtual clinic patients.

**Figure 4:**
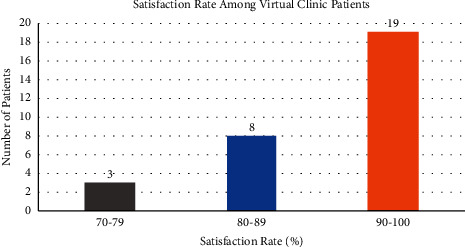
Satisfaction rate among virtual clinic patients.

The virtual TKR clinic included 30 post-TKR surgery patients (Table 1). The average follow-up period was 39.1 months (SD 17.07). The earliest follow-up is 20 months after TKR surgery; meanwhile, the longest follow-up is 97 months after TKR surgery. Most of the patients have an excellent score, with an average OKS of 45 (Figure 2) and a motion arc of more than 120° (Figure 3). Only one patient has a score below 40. The average consultation time for the virtual clinic is 9 minutes 21 seconds, with the longest consultation time being 10 minutes 44 seconds and the shortest consultation time being 7 minutes. Most of the patients were satisfied with the online consultation, with mostly the satisfaction rate above 80%, and only two patients had a satisfaction score below 80% (Figure 4).

### 3.2. Discussion

By the time the study was conducted, the number of COVID-19 cases in Indonesia was increased (average: 5,600 new cases daily). The preexisting healthcare infrastructure back then was still developing to deal with the rise of COVID-19 cases, such as the limited capacity of facilities and human resources. The healthcare service was primarily focused on COVID-19 service, since the number of COVID-19 cases was increasing. Thus, the increasing number of COVID-19 cases has greatly impacted the healthcare system in the Nation, including orthopedic services. Almost all aspects of the orthopedic field, such as emergency unit, outpatient clinic, inpatient unit, and elective surgery, have been significantly altered [9, 10]. In May 2021, the Jakarta Government imposed microscale public activity restrictions (Pemberlakuan, Pembatasan, Kegiatan, Masyarakat, Berskala, Mikro/PPKM Mikro) to decrease the number of COVID-19 cases in Jakarta [11, 12]. Due to the implementation of the new rule, people from other cities were prohibited to go to Jakarta without special permission from the government. This made some of the patients who resided outside the Jakarta area not able to come to the hospital for their routine follow-up. For this reason, the virtual clinic for TKR patients' follow-up was initiated.

Furthermore, most of the patients who underwent TKR surgery are geriatric patients with comorbidities. According to the World Health Organization (WHO) and Center for Disease Control and Prevention (CDC), the elderly ones are more susceptible to COVID-19 infection and they face a higher risk of developing severe illness [13, 14]. Indonesian Doctor Association (Ikatan Dokter Indonesia (IDI)) recommended the elderly not to visit hospitals unless in an emergency case [15]. The virtual clinic can also reduce the volume of outpatient clinics to support physical distancing [16]. The patients who were asked to participate in the virtual clinic are patients whose surgeries are more than 24 months because usually in post-TKR patients, prescription medicine after 2 years is not essential anymore [17].

A single-center study conducted in a trauma center in one of Italy's biggest COVID-19 hospitals during the COVID-19 period by De Mauro et al. [18] concluded that there were six patients with COVID-19 who underwent orthopedic surgery procedures and all of them did not have any complication intraoperatively and postoperatively, with a standardized safety protocol used during and following the surgery. Nonetheless, this study also stated that a strict safety measure needs to come first, seeing that the majority of the orthopedic patient is elderly and, thus, more vulnerable to the COVID-19 infection. This is also the reason why we conducted the virtual clinic in the first place for the TKR follow-up, as a postoperative preventive measure to minimize the risk.

In order to reduce the risk in those with comorbidities, there was also another study by Romeo et al. [19] about the effectiveness of single use instrument (SUI) for TKR. They stated that SUI has increased the sterility of operating theatre and decreased the risk of prosthetic joint infection. The use of SUI has been shown to reduce the incidence of prosthetic joint infection (PJI), and PJI is one of the most common cause of revision surgery. Thus, especially for patients with comorbidities, the use of SUI is beneficial in order to avoid the risk of acquiring PJI following the surgery and lower the need for revision surgery.

Studies have reported the benefits and roles of continued telehealth utilization past the COVID-19 pandemic, including improved access, improved communication with patients undergoing surgical treatment, and improved reporting of functional outcomes in research settings [20, 21] Grandizio et al. [22] found that telemedicine resulted in high levels of patient satisfaction, decreased visit times, and decreased travel burdens compared with conventional in-person appointments.

To the best of our knowledge, the current study is the first study describing the use of telemedicine as a follow-up tool for TKR surgery. This study shows that the use of telemedicine in orthopedic patients, particularly in arthroplasty patients, can be an alternative to a face-to-face clinic, especially during the COVID-19 pandemic. The virtual follow-up clinic can also be used in other orthopedic subspecialties [23]. Despite the mentioned strengths, the study also has its limitations concerning the number of patients included in this study. Also, the virtual clinic as the alternative for postoperative follow-up cannot be used for the long term since majority of patients were geriatric with some comorbidities who need special care. Nevertheless, the virtual clinic can serve as an effective option in cost-reduction, especially in the midst of the COVID-19 pandemic [24].

## 4. Conclusions

The current study showed that a virtual clinic for knee arthroplasty serves as a promising and effective alternative to a face-to-face clinic assessment. The results showed a high satisfaction rate during the COVID-19 pandemic, which can potentially expand to the postpandemic era.

## Figures and Tables

**Table 1:** Patients' follow-up results from the virtual clinic.

No.	Follow-up period (months)	OKS (0–19 poor; 20–29 moderate; 30–39 good; 40–48 excellent) (points)	OKS interpretation	ROM flexion (degree)	Satisfaction rate (%)
1	43	46	Excellent	135	90
2	53	47	Excellent	135	92
3	40	42	Excellent	135	82
4	41	48	Excellent	135	100
5	33	46	Excellent	135	90
6	97	46	Excellent	135	96
7	39	46	Excellent	135	90
8	41	47	Excellent	135	92
9	24	47	Excellent	100	92
10	30	42	Excellent	120	82
11	33	41	Excellent	100	80
12	33	46	Excellent	135	90
13	28	40	Excellent	120	78
14	39	47	Excellent	135	92
15	38	46	Excellent	135	90
16	27	44	Excellent	120	86
17	20	44	Excellent	135	86
18	94	44	Excellent	110	86
19	38	46	Excellent	110	90
20	30	48	Excellent	110	100
21	28	38	Good	110	74
22	29	40	Excellent	110	78
23	40	48	Excellent	135	100
24	34	43	Excellent	135	84
25	44	48	Excellent	130	94
26	29	48	Excellent	130	94
27	28	47	Excellent	120	92
28	52	48	Excellent	135	94
29	31	48	Excellent	135	94
30	24	45	Excellent	135	87

## Data Availability

The data used to support the findings of this study are included within the article.
